# Effects of a culturally adapted group based Montessori based activities on engagement and affect in Chinese older people with dementia: a randomized controlled trial

**DOI:** 10.1186/s12877-020-01967-0

**Published:** 2021-01-07

**Authors:** Helen Yue-lai Chan, Yee-man Yau, Si-fan. Li, Ka-shi Kwong, Yuen-yu Chong, Iris Fung-kam Lee, Doris Sau-fung Yu

**Affiliations:** 1 The Nethersole School of Nursing, Faculty of Medicine, The Chinese University of Hong Kong, Hong Kong SAR, China; 2 Social Services Department, The Salvation Army, Hong Kong and Macau Command, Hong Kong SAR, China; 3 Nethersole Institute of Continuing Holistic Health Education, Hong Kong SAR, China; 4 School of Nursing, Faculty of Medicine, The University of Hong Kong, Hong Kong SAR, China

**Keywords:** Dementia, Person-centered, Group activity, Community, Support

## Abstract

**Background:**

The Montessori Method underpinned by the principle of person-centered care has been widely adopted to design activities for people with dementia. However, the methodological quality of the existing evidence is fair. The objectives of this study are to examine the feasibility and effects of a culturally adapted group-based Montessori Method for Dementia program in Chinese community on engagement and affect in community-dwelling people with dementia.

**Methods:**

This was a two-arm randomized controlled trial. People who were aged 60 years or over and with mild to moderate dementia were recruited and randomly assigned to the intervention group to receive Montessori-based activities or the comparison group to receive conventional group activities over eight weeks. The attendance rates were recorded for evaluating the feasibility. The Menorah Park Engagement Scale and the Apparent Affect Rating Scale were used to assess the engagement and affect during the activities based on observations. Generalized Estimating Equation model was used to examine the intervention effect on the outcomes across the sessions.

**Results:**

A total of 108 people with dementia were recruited. The average attendance rate of the intervention group (81.5%) was higher than that of the comparison group (76.3%). There was a significant time-by-group intervention effect on constructive engagement in the first 10 minutes of the sessions (Wald χ^2^ = 15.21–19.93, *ps* = 0.006–0.033), as well as on pleasure (Wald χ^2^ = 25.37–25.73, *ps* ≤ 0.001) and interest (Wald χ2 = 19.14–21.11, *p*s = 0.004–0.008) in the first and the middle 10 minutes of the sessions, adjusted for cognitive functioning.

**Conclusions:**

This study provide evidence that Montessori-based group activities adapted to the local cultural context could effectively engage community-dwelling Chinese older people with mild to moderate dementia in social interactions and meaningful activities and significantly increase their positive affect.

**Trial registration:**

ClinicalTrials.gov, NCT04352387. Registered 20 April 2020. Retrospectively registered.

## Background

Most of the people with dementia exhibit behavioural and psychological symptoms of dementia (BPSD), for example agitation, repetition, restlessness, wandering, apathy and depressed mood, resulting from cognitive impairment [1, 2]. Non-pharmacological interventions have been advocated as the first line management for addressing the modifiable factors, and thereby reducing the use of anti-psychotic or sedative medications to prevent the associated adverse effects and mortality risk, and improve the quality of life of people with dementia [3–6]. The effects of non-pharmacological interventions, for example, multisensory stimulation, cognitive or emotion-oriented interventions, behavioral management strategies, and physical exercise, have been widely studied for matching the unmet psychosocial need for sensory deprivation, social interaction and meaningful activities in people with dementia [4, 6, 7]. While systematic reviews concluded that the effects of these non-pharmacological interventions on managing challenging behaviors are inconsistent, it is widely agreed that strategies that only passively engage people with dementia have poor effects, whereas those personalized to individual needs are more promising [1, 5–7].

The Montessori Method has been adopted to design activities for people with dementia over the past two decades [8, 9]. This method was originally developed by Dr Maria Montessori, a physician and educator, in the early 1900s as an educational approach for training children with functional skills for practical life challenges through sensorial experiences in the everyday environment. Montessori programming highlights task breakdown, guided repetition, progression in task difficulty from simple to complex or concrete to abstract, the careful matching of individual past interests and occupations, and self-correcting, all aligned with the concept of rehabilitation [8, 10, 11]. Dr Cameron Camp and his associates proposed the Montessori Method as a strength-based approach for creating personalized interventions in dementia care to maximize the spared capacity and abilities of each individual [8]. The activities in the Montessori-based for dementia (MMD) program highlights the importance of considering the individual’s past experiences and interests [9, 10]. The essence of these activities is consistent with the person-centered care approach recommended for dementia care [12, 13]. The acronym “CREATE” is used to represent the principles of activity design, they are: Create a prepared environment, Remove unnecessary distractions, Error free process, All materials are familiar to the participants in real life setting, Templates and manipulatives are provided according to individual needs and ability, and Environment is prepared in home-like. Generally speaking, the activities aim to engage participants in meaningful activities and, through which, promote learning through procedural or implicit memory with the support of prepared environment and external cues [9, 10]. Thus far, Montessori-based activities for dementia have been conducted in a variety of formats, such as Memory Bingo or sorting pictures or words into categories, fine-motor tasks such as cutting and stringing beads, reading groups, the use of templates, and the creation and production of certain products, on an individual or group basis [10, 11]. While activities conducted on a one-to-one basis may allow more flexibility in activity design and greater attention for people with dementia, literature suggests that group format creates opportunities for people with dementia to take up meaningful social roles in the group that allow them to use their social skills, and promotes peer interaction and learning [8, 14, 15].

Literature generally suggested that Montessori-based activities increased constructive engagement in people with dementia [3, 11, 14, 16, 17] but there were mixed results on positive and negative affect [11, 18, 19]. Moreover, the practices of conducting Montessori-based activities varied widely [3] and were mainly conducted on individual format [11]. Previous studies which examined the effects of Montessori-based activities in the Chinese people with dementia focused on agitation and were only conducted in care home setting [20, 21]. Moreover, the methodological quality of these existing studies were limited by single subject design and lack of randomization [3, 11, 17, 19, 20]. The purposes of this study were to evaluate the feasibility of culturally adapted group-based Montessori-based activities in the Chinese communities and examine the effects of these activities on the type and level of engagement and affect of Chinese older people with mild to moderate dementia.

## Methods

### Participants

This was a multicenter two-arm parallel randomized controlled trial design conducted in three community centers, three day care centers and three care homes operated by The Salvation Army in Hong Kong between August 2018 and July 2019. The study adhered to the CONSORT guidelines. Participants were randomly assigned either the intervention group or the comparison group on 1:1 ratio by stratified randomisation based on the study site given that the health conditions of the older adults in different care settings might greatly vary. The random sequence was based on computer-generated lists using a block size of six within strata to ensure a balance allocation. A researcher who was not involved in subject recruitment and intervention design was responsible for the random allocation. The inclusion criteria were care recipients at the study venues aged 60 years or above, with a diagnosis of dementia at a stage 3 to 5 on the global deterioration scale (GDS) [20, 22], and were not joining any other dementia training activities. They were excluded if they presented a hazard to others through disruptive behaviors or proxy consent could not be obtained. The sample size was estimated based on the level of constructive engagement during the activity. According to Judge et al.’s study (2000) [14], the effect size of the Montessori-based activities in people with dementia were approximately 1.5. With an estimated 20% attrition rate, 18 participants will be needed for each arm for a two-sided test with a Type I error of 0.05 at 80% power. The overall sample size was 108, with a subsample of 36 participants from each type of setting given the context of the three types of care settings may vary.

### Description of the intervention

Participants in the intervention group received MMD program whereas participants in the comparison group received conventional group activities provided at the study venues on different days over eight weeks. All sessions were conducted in group format with about six participants per group for one hour. The group format created opportunities for participants to express their social skills during the activities [12]. The program design covered five aspects, namely cognitive stimulation, life skills, motor movements and fitness, sensory stimulation, and socialization. Each 1-hour session included two to three activities tailored to the local cultural context. A standardized manual and activity materials were developed to ensure the treatment fidelity. The program was delivered by staff who had completed certified training in Montessori-based activities. Participants in the comparison group received activities delivered regularly in the usual care, such as reading out newspapers, physical activities, and watching videos, in the sessions delivered by staff members who were blinded to the study design and had not received training in Montessori-based activities.

### Study outcomes

The primary outcome was type and level of engagement and the secondary outcome was affect during the activities. Observations were conducted on the individual participant for a ten-minute period at the beginning, middle, and end of the activity of each session. The Menorah Park Engagement Scale (MPES) was used to measure engagement in terms of constructive engagement, passive engagement, non-engagement and other engagement in activities [14]. The level of engagement was determined based on observation and rated on a three-point Likert scale, from 0 = not at all observed to 2 = equal or more than half of the observed time. MPES was found to be sensitive to measure the effects of activity programming of Montessori-based activities [23]. The Apparent Affect Rating Scale (AARS) was used to measure five positive and negative affect, including pleasure, anger, anxiety/fear, depression/sadness, and interest, through observation [24]. Each affect was measured on a five-point Likert scale, from 1 = never to 5 = more than five minutes. It is a valid and reliable measure of affect in care home residents with dementia and has been used to measure Montessori-based activities in previous studies [11]. Demographic data, including age, sex, level of GDS and score of Montreal Cognitive Assessment (MoCA), were collected at baseline.

### Data collection

Ethical approval for the study was obtained from the Survey and Behavioural Ethics Committee of The Chinese University of Hong Kong (no reference number is available). Staff members of the participating venues initially screened all the clients and approached the designated family member of the potential participants for recruitment. Proxy consent to participate in the study was obtained in a written format from the family member who was the primary carer of the care recipients who met the study inclusion criteria. They were asked to provide consent of allowing their relatives with dementia, i.e. the potential participants, to participate in either the MMD program or the usual activities as scheduled and being videotaped during the sessions to facilitate observation of their reactions and behaviours. In addition to verbal explanation, an information sheet about the study purpose and the nature of the data collection were provided for consideration. All data were coded and used on an anonymous basis. All sessions were videotaped for research purpose only to facilitate assessment and were not disclosed or shared publicly. Assent to join the activities was also obtained from the potential participants each time before the session. Participation was voluntary. Two research assistants with psychology backgrounds and blinded to the group allocation watched the videos independently to conduct the observations. They record the length of time for the different types of engagement and affect exhibited by each participant during the session. Training on the data collection was provided. The inter-rater reliability in study outcomes was established with over 90% agreement for 30 observations between the research assistants to ensure consistency of assessment. Attendance record were kept for all sessions to evaluate the feasibility. A phone follow up was conducted after each session with those who were absent to clarify the reasons.

### Statistical analysis

The intention to treat principle was applied in the analysis [25]. Descriptive statistics were used to summarize the participants’ socio-demographic data, cognitive state and the study outcomes. Independent t test and Chi-square test were used to compare the homogeneity between the intervention group and the comparison group at baseline, as appropriate. A Generalized Estimating Equation model was used to examine the intervention effect on the study outcomes between groups across eight sessions, adjusting for covariates. SPSS (version 25.0 IBM, Armonk, NY, US) was used for the statistical analysis.

## Results

### Participants’ characteristics

**Fig. 1:**
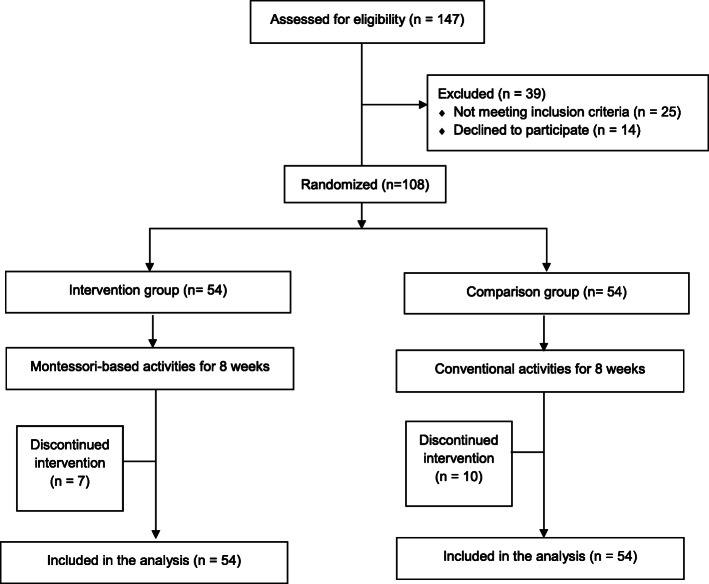
CONSORT diagram

As shown in Fig. 1, a total of 147 participants were screened for eligibility for the study. Of the 122 potential participants, 108 participated in the study. The major reasons for not participating in the study were lack of interest in the study, inability to attend the sessions due to mobility problems or a lack of escort services, and inability to obtain proxy consent from family members. Table 1 shows the participants’ characteristics. Their mean age was 83.9 (SD 7.0) years, ranging from 67 to 99. Female participants accounted for 71.0% of the total. No significant differences in the demographic and clinical characteristics were observed between the two groups at baseline.

**Table 1:** Participants’ characteristics

	Comparison Group (*n* = 54)	Intervention Group (*n* = 54)	*p* value^*^
Age (years), mean (SD)^a^	84.5 (6.5)	83.3 (7.5)	0.363
Sex, *n* (%) Male Female	16 (29.6) 38 (70.4)	17 (31.5) 37 (68.5)	0.828
Global Deterioration Scale, *n* (%)			0.270
Stage 3	5 (9.3)	4 (7.4)	
Stage 4	32 (59.3)	35 (64.8)	
Stage 5	17 (31.5)	15 (27.8)	
MoCA scores, mean (SD)a	8.9 (5.0)	9.1 (5.5)	0.882

**Notes**: ^*^Chi-square test, unless specified; ^a^independent t test; *SD* standard deviation; *n* number

### Feasibility

The mean attendance rate of the sessions was 79.1%. The average attendance rate of the intervention group (81.5%) was higher than that of the comparison group (76.3%), but the difference was not statistically significant. The common reasons for non-attendance were time clash with medical appointment (11.1%), not interest in the activities (7.4%), change in health condition and hospitalization (2.8%).

### Engagement

The effects of MMD program on the types and levels of engagement between the intervention group and the comparison group across the treatment sessions is shown in Table 2. The intervention group was found to have a significantly higher level of constructive engagement in the first 10 minutes of each session (Wald chi-square = 15.2, *p* = 0.033) and in the middle 10 minutes of each session (Wald chi-square = 19.9, *p* = 0.006), as well as lower level of passive engagement in the last 10 minutes of each session (Wald chi-square = 17.61, *p* = 0.014) across the study period, after adjusting for MoCA score and GDS score. Subgroup analysis revealed no differences between participants in the community care and those in the care home settings.

**Table 2:** Effects of MMD program versus usual care on the MPES scores across treatment sessions

Measures	Time effect		Group effect		Time-by-group effect	
	Wald χ^2^	*p*	Wald χ^2^	*p*	Wald χ^2^	*p*
***Constructive engagement***						
At the beginning 10 minutes of the session						
Intervention group	28.75	< 0.001***	20.07	< 0.001***	15.21	0.033*
Comparison group						
At the middle 10 minutes of the session						
Intervention group	13.26	< 0.001***	30.15	< 0.001***	19.93	0.006**
Comparison group						
At the last 10 minutes of the session						
Intervention group	2.79	NS	36.94	< 0.001***	3.54	0.831
Comparison group						
***Passive engagement***						
At the beginning 10 minutes of the session						
Intervention group	21.85	0.003**	0.41	NS	5.40	0.612
Comparison group						
At the middle 10 minutes of the session						
Intervention group	2.34	NS	4.79	0.029*	7.96	0.336
Comparison group						
At the last 10 minutes of the session						
Intervention group	7.56	NS	9.45	0.002**	17.61	0.014*
Comparison group						
***Non-engagement***						
At the beginning 10 minutes of the session						
Intervention group	7.28	NS	7.28	0.007**	8.67	0.277
Comparison group						
At the middle 10 minutes of the session						
Intervention group	4.65	NS	7.26	0.007**	2.04	0.958
Comparison group						
At the last 10 minutes of the session						
Intervention group	11.17	NS	6.43	0.011*	5.32	0.621
Comparison group						
***Other engagement***						
At the beginning 10 minutes of the session						
Intervention group	15.30	0.032*	2.45	NS	9.27	0.234
Comparison group						
At the middle 10 minutes of the session						
Intervention group	13.30	NS	0.16	NS	12.32	0.090
Comparison group						
At the last 10 minutes of the session						
Intervention group	7.90	NS	0.65	NS	12.44	0.087
Comparison group						

**Note**: Generalized estimating equation; Wald χ^2^ = Wald Chi-square; *NS* not significant; *p < 0.05, ** *p* < 0.01, ***p < 0.001

### Affect

Only two aspects of affect measured by the AARS, pleasure and interest, were included in the analysis because anxiety/fear and sadness were rarely observed. As shown in Table 3, there was a significant time-by-group intervention effect on pleasure in the first 10 minutes of each session (Wald chi-square = 25.4, *p* < 0.001) and in the middle 10 minutes of each session (Wald chi-square = 25.7, *p* < 0.001) across the entire session period, as well as on interest in the first 10 minutes of each session (Wald chi-square = 21.1, *p* = 0.004) and in the middle 10 minutes of each session (Wald chi-square = 19.1, *p* = 0.008), by adjusting the MoCA score and the GDS score. No differences were noted between participants in the community care and those in the care home settings in the subgroup analysis.

**Table 3:** Effects of MMD Program versus usual care on AARS scores across treatment sessions

Measures	Time effect		Group effect		Time-by-group effect	
	Wald χ^2^	*p*	Wald χ^2^	*p*	Wald χ^2^	*p*
***Pleasure***						
At the beginning 10 minutes of the session						
Intervention group	13.08	NS	20.97	< 0.001***	25.37	< 0.001***
Comparison group						
At the middle 10 minutes of the session						
Intervention group	7.34	NS	35.19	< 0.001***	25.73	< 0.001***
Comparison group						
At the last 10 minutes of the session						
Intervention group	3.41	NS	33.51	< 0.001***	13.18	NS
Comparison group						
***Interest***						
At the beginning 10 minutes of the session						
Intervention group	16.92	NS	8.56	0.003**	21.11	0.004**
Comparison group						
At the middle 10 minutes of the session						
Intervention group	9.52	NS	1.19	NS	19.14	0.008**
Comparison group						
At the last 10 minutes of the session						
Intervention group	12.94	NS	1.89	NS	11.09	NS
Comparison group						

**Note**: Generalized estimating equation; Wald χ^2^ = Wald Chi-square; *NS* not significant; *p < 0.05, ** *p* < 0.01, ***p < 0.001

## Discussion

This study evaluates the feasibility and effectiveness of a culturally adapted group based MMD program on engagement and affect in older people with mild to moderate dementia in the Chinese communities. MMD program is a strength-based approach intervention highlighting the importance of tailoring personalized activities according to individual’s interests and abilities [3, 8, 9]. The experience from this study suggested that the Montessori-based activities were acceptable to the community-dwelling Chinese older people with dementia in both the community care setting and care home setting. Participants in the intervention group exhibited more constructive engagement during the activities than did the comparison group. Also, less passive engagement was observed in the intervention group compared to the comparison group. These findings echo the findings of previous studies that higher level of constructive engagement and lower level of passive engagement or non-engagement were observed during Montessori-based activities [3, 14, 18, 26]. This study confirms that the personalized activities adapted to the local cultural context could increase constructive engagement and reduce passive engagement among people with dementia in Chinese communities [5, 13].

The findings also suggest that the MMD program increase positive affect in older Chinese people with mild to moderate dementia. Higher levels of pleasure and interest were observed during the Montessori-based activities in the intervention group across sessions than the conventional activities in the comparison group. This is consistent with previous studies that Montessori based activities conducted on individual basis can increase positive affect and interest in people with dementia [11, 19, 26]. This study demonstrated that the Montessori-based activities in a small group format is also effective in increasing positive affect in people with dementia. Lin and associates (2009) compared the effects of Montessori-based activities with acupressure noted that the increased in positive affect in participants joining the Montessori-based activities may be due to the social nteractions among group members during the activities [27].

Despite the merits of the MMD program, more preparatory works and manpower was needed compared with other conventional activities as the staff members need to explore the uniqueness of each individual and maintain close observation so as to modify the activity or materials to meet individual’s characteristics and group dynamics. We acknowledged several study limitations when interpreting the findings. First, this study included a small sample size from community care and care home settings. Further research should include a larger sample size to increase the generalizability of the findings. Second, the study outcomes concerned about the participants’ engagement and affect reflected the immediate effects of the intervention only. Follow-up assessment over a longer period is warranted to examine the sustained effects of the program. Third, the influence of other socio-demographic factors on the study outcomes were not examined. In addition, assessment about its effects on the participants’ cognitive functioning and behaviors beyond the group sessions, and costs related to the intervention implementation should also be explored in future studies.

## Conclusions

This study used a robust study design to evaluate the effects of a culturally specific Montessori-based activities in Chinese community-dwelling older people with mild to moderate dementia. The design of these activities valued individual’s past experiences and present abilities, highlighting the importance of person-centered care. The evidence supports that such approach is feasible and effective in increasing the level of engagement and positive affect of people with dementia through meaningful activities and social interactions.

## Data Availability

The datasets used and/or analysed during the current study are available from the corresponding author on reasonable request.

## Abbreviations

AARS: Apparent Affect Rating Scale
MMD: Montessori Method for Dementia
GDS: Global Deterioration Scale
MPES: Menorah Park Engagement Scale
MoCA: Montreal Cognitive Assessment
